# Abatacept in rheumatoid arthritis-associated interstitial lung disease: short-term outcomes and predictors of progression

**DOI:** 10.1007/s10067-021-05854-w

**Published:** 2021-07-27

**Authors:** Marika Tardella, Marco Di Carlo, Marina Carotti, Andrea Giovagnoni, Fausto Salaffi

**Affiliations:** 1 Rheumatology Clinic, Università Politecnica Delle Marche, Via Aldo Moro, 25 - 60035 – Jesi, Ancona, Italy; 2 Department of Radiology, Ospedali Riuniti, Università Politecnica Delle Marche, Ancona, Italy

**Keywords:** Abatacept, High-resolution computed tomography, Interstitial lung disease, Rheumatoid arthritis

## Abstract

**Introduction:**

Interstitial lung disease in rheumatoid arthritis (RA-ILD) is an extra-articular involvement that impairs the prognosis and for which there is still no well-coded treatment. The aim of this study was to evaluate abatacept (ABA) effectiveness and safety in patients with RA-ILD.

**Methods:**

RA-ILD patients who started ABA treatment were consecutively enrolled. Chest high-resolution computed tomography (HRCT), clinical, laboratory and respiratory function variables were collected at baseline and after 18 months of ABA treatment. HRCT abnormalities were evaluated using a computer-aided method (CaM). ABA response was established based on the change in the percentage of fibrosis evaluated at HRCT-CaM, dividing patients into “worsened” (progression ≥ 15%), “improved” (reduction ≥ 15%), and “stable” (changes within the 15% range). The multivariate regression model was used to assess the associations between RA characteristics and ABA response.

**Results:**

Forty-four patients (81% women, mean age 59.1 ± 8.0, mean disease duration of 7.5 ± 3.1 years) were studied. Five patients (11.4%) showed RA-ILD progression, 32 patients (72.6%) were considered stable, and 7 patients (16.0%) showed an RA-ILD improvement. The proportion of current smokers was significantly different between “worsened” patients, respect to those defined as "improved/stable” (p = 0.01). Current smoking habit (p = 0.005) and concomitant methotrexate treatment (p = 0.0078) were the two variables related to RA-ILD progression in multivariate regression analysis.

**Conclusion:**

Treatment with ABA is associated with a RA-ILD stability or improvement in the 88.6% of patients. Current smoking habit and concomitant treatment with methotrexate are the modifiable factors associated with RA-ILD worsening.

**Table:** 

**Key Points** *• Abatacept plays a favourable role in the control of RA-ILD, with a significant worsening in only 11.4% of patients during a 18-month follow-up period.* *• The predictive variables related to RA-ILD progression during abatacept therapy are the concomitant treatment with methotrexate and current smoking habit. *

## Introduction

Rheumatoid arthritis (RA) is a progressive systemic autoimmune disorder characterized by articular and extra-articular manifestations affecting about 0.5% of the adult population in Western countries [1]. Interstitial lung disease (ILD) is one of the most important extra-articular manifestations in RA [2]. The prevalence of RA-ILD varies from 1 to 67% depending on the method used to assess lung involvement and the study design [3–6]. The most commonly associated risk factors for predicting RA-ILD are advanced age, old age at onset of RA, male gender, smoking status and presence of anti-citrullinated peptide antibodies (ACPA) [7, 8]. In addition, some effective drugs used to treat RA can cause lung toxicity [9].

High-resolution computed tomography (HRCT) of chest provides valuable information about ILD, including the pattern and extent of the disease [10]. HRCT abnormalities are found in 48–68% of asymptomatic patients and 90% of symptomatic patients with RA [11, 12]. During an average follow-up of 1.5 years, up to 57% of patients with asymptomatic RA-ILD have experienced a HRCT progression [13]. The usual interstitial pneumonia (UIP) pattern is more frequent in men and is associated with a worse prognosis, while the non-specific interstitial pneumonia (NSIP) pattern is more related to the female gender and has a better prognosis [14, 15]. The 5-year survival rate is 36% in patients with RA-ILD-UIP and 94% in patients with RA-ILD-NSIP, confirming the favourable outcome of patients with  this last pattern [14].

This scenario highlights the need for effective treatment for RA-ILD, but its management is still debated and somewhat controversial [16]. In addition, the pulmonary toxicity of some disease-modifying anti-rheumatic drugs (DMARDs), particularly methotrexate (MTX), is still debated [17]. Immunosuppressive treatments also increase the risk of infection and, in particular, of severe lung infection with a high rate of hospitalization. On the other hand, certain biologic DMARDs (bDMARDs) demonstrated a promising effectiveness in slowing or stopping the progression of RA-ILD. Among these, abatacept (ABA), a T lymphocyte co-stimulation antagonist used in the treatment of RA, has shown some efficacy in the treatment of RA-ILD. ABA is also promising in light of the reduced infectious risk if compared to other bDMARDs [18]. However, the number of studies published on this issue is still small and mostly retrospective [19–21].

Therefore, the main aim of this study was to evaluate the efficacy and safety of ABA treatment in RA-ILD patients and, as a second aim, to identify predictors of an unfavourable treatment outcome.

## Methods

### Study population and assessment

This study included patients with a diagnosis of RA according to the American College of Rheumatology/European League Against Rheumatism classification criteria [22] and with a coexisting diagnosis of ILD, according to the criteria of the American Thoracic Society/ATS/ERS 2015 [23].

From January 2016 to December 2019, RA-ILD patients attending the outpatient and inpatient clinics of the Rheumatology Clinic of the Polytechnic University of Marche (Italy) were consecutively enrolled. Inclusion criteria were the presence of RA-ILD and the need to start biotechnological drug for active RA, refractory to current therapy, if present. Patients who were concomitantly receiving MTX or other conventional synthetic DMARDs (csDMARDs) and/or glucocorticoids with a dosage of less than 10 mg/day prednisone or equivalent were included. We also included RA patients previously treated with bDMARS, discontinued due to intolerance or ineffectiveness. Patients with a history of pulmonary disease except for ILD, active malignancy, chronic heart failure and previous treatment with ABA were excluded.

ABA treatment was started at a dose of 125 mg/week subcutaneously for all patients, and this time was considered as time zero of the study. Patients were then followed according to normal daily practice with semiannual outpatient visits. A trained nurse monitored the administration of the drug and the occurrence of adverse events weekly by phone or e-mail.

Baseline data were collected by a rheumatologist at time zero and included demographic variables, smoking habits, disease duration (defined as time since RA diagnosis), concomitant therapies and assessment of disease activity (Clinical Disease Activity Index (CDAI) [24] and Health Assessment Questionnaire-Disability Index (HAQ-DI) [25]). The presence of rheumatoid factor (RF) and ACPA was recorded. On the same day, pulmonary symptoms were assessed using the modified Borg Dyspnoea Index (BDI) [26].

Pulmonary function test (PFT), single-breath diffusion lung capacity of carbon monoxide (DLCO, % predicted, corrected for haemoglobin) and HRCT were performed within 2 weeks of the starting of ABA and after 18 months. Patients who discontinued ABA due to intolerance or ineffectiveness (CDAI persistently higher than 14) or who did not undergo HRCT after 18 months were excluded from the study. The diagnosis of ILD was performed using chest HRCT, and a quantitative evaluation of pulmonary fibrosis was performed using a computerized method of quantification (CaM), based on what has been described in detail in previous works [27–29]. HRCT images were reconstructed and analysed by OsiriX MD 7, a DICOM visualization software (OsiriX MD version 7, 64-bit format) on a Mac Mini (2.8 GHz Intel Core 2 Duo Desktop Computer, 16 GB random access memory; Apple Computer, Cupertino, CA, USA) with Mac OSX 10.12.2 operating system. Lung parenchymal abnormalities on HRCT were coded and evaluated by two independent radiologists, expert in lung diseases and blinded to the clinical data, using the CaM quantification process. No patient underwent lung biopsy.

HRCT examination was repeated at 18 months after time zero, i.e. initiation of ABA treatment. This examination was also assessed semiquantitatively with CaM.

The local Ethics Committee (Comitato Unico Regionale—ASUR Marche, No 2015 0458 AS) approved the protocol. The study was conducted in accordance with the Helsinki Declaration in its fifth edition (2000). All patients signed the informed consent.

### Statistical analysis

Data were recorded in a Microsoft Excel database and processed with MedCalc 19.0.6 (statistical software packages for Windows XP). The Kolmogorov–Smirnov test was used to verify the normal distribution. Where appropriate, the medians and interquartile ranges (IQR), as well as the means and standard deviations (SD), are presented.

A parametric two-sample t test and one-way analysis of variance (ANOVA) test were used to compare continuous variables and the χ2 test to compare categorical variables between patients. A two-sided coupled t test and the non-parametric Wilcoxon signed rank test were used to compare values at baseline and after 18-months of follow-up.

The analyses of the chest HRCT investigations performed at baseline and after 18 months were conducted dividing patients into three groups on the basis of the CaM-HRCT progression: patients with a lung fibrosis progression ≥ 15% were defined as “worsened”, those with a reduction of ≥ 15% were defined as “improved”, all other patients were defined as “stable”. The 15% CaM variation threshold resulted from the determination of the standard deviation of the mean value variation after 18 months of follow-up.

Finally, we performed multivariate corrected regression analysis in order to assess the strength of the association between RA characteristics at baseline and HRCT response to ABA. The quantification of CaM was considered as dependent variable. The covariates included age, sex, disease duration, age at disease onset, smoking habit, RF presence, ACPA presence, CDAI and HAQ-DI. The results were expressed as multivariate regression coefficient (R) and corrected square regression coefficient (R^2^) for the number of variables included in the analysis. This allows to calculate the predictivity of each multivariate model based on the number of variables inserted in the model. The significance has been set to p < 0.05.

## Results

Fifty-four patients were included at time zero: 10 (18.5%) patients were eliminated during the course of the study, of whom 4 patients experienced a minor adverse event (3 for skin rush and one patient for diarrhoea) and 6 patients for ineffectiveness of ABA after 6 months of treatment (CDAI persistently higher than  22). No severe adverse events or deaths were reported in the followed cohort.

We therefore analysed the data of 44 patients (81% women) who completed the study. The mean age was 59.1 ± 8.0 years, and the mean disease duration was 7.5 ± 3.1 years. Twenty-three (52.3%) patients were ACPA positive and 28 (63.6%) RF positive. At baseline the percentage of current smokers was 38.6%. Mean clinical and instrumental data at time zero are summarised in Table 1. All patients were concomitantly treated with csDMARD, in particular MTX (20 patients, 45.5%), hydroxychloroquine (10 patients, 22.7%), leflunomide (8 patients, 18.2%), sulfasalazine (6 patients, 13.6%) at time zero. Sixteen (36.4%) patients were previously treated with a bDMARD, including etanercept (6 patients, 13.6%), adalimumab (6 patients, 13.6%), and tocilizumab (4 patients, 9.2%). A total of 31 (70.4%) patients were treated with corticosteroids at a mean dose of 3.7 (range 1.25–8.5) mg prednisolone/day equivalent. No patients developed tuberculosis during ABA therapy. Four patients underwent prophylactic antituberculous therapy in the month prior to inclusion in the study due to QuantiFERON test positivity.

**Table 1:** Demographic characteristics, disease activity, functional disability, lung function, and high-resolution computed tomography data at the baseline (T0) and after 18 months of treatment (T18), expressed in means and standard deviations

	T0		T18		
	Mean	SD	Mean	SD	p*
Age (years)	59.05	8.03	–	-	
Disease duration (years)	7.55	3.09	–	-	
CDAI	34.66	10.05	10.11	7.58	< 0.001
HAQ-DI	1.45	0.32	0.75	0.29	< 0.001
Borg Dyspnea Index	2.54	1.23	1.90	1.01	0.01
DLco (% predicted)	58.69	8.24	61.26	11.23	0.22
FVC (% predicted)	82.29	4.86	81.24	11.97	0.59
HRCT-CaM fibrosis (percentage)	19.41	5.89	18.94	6.06	0.71

Legend and abbreviations: * = two-sided paired Student t test; SD = standard deviation; CDAI = Clinical Disease Activity Index; HAQ-DI = Health Assessment Questionnaire Disability Index; DLco = diffusion lung capacity of carbon monoxide; FVC = forced vital capacity; HRCT = high-resolution computed tomography; CaM = computer-aided method.

The patients experienced a significant improvement in RA disease activity and joint function. The mean CDAI score decreased from 34.66 to 10.11 (p < 0.001), and the mean HAQ-DI score decreased from 1.45 to 0.75 (p < 0.001) at 18 months (Table 1).

**Fig. 1:**
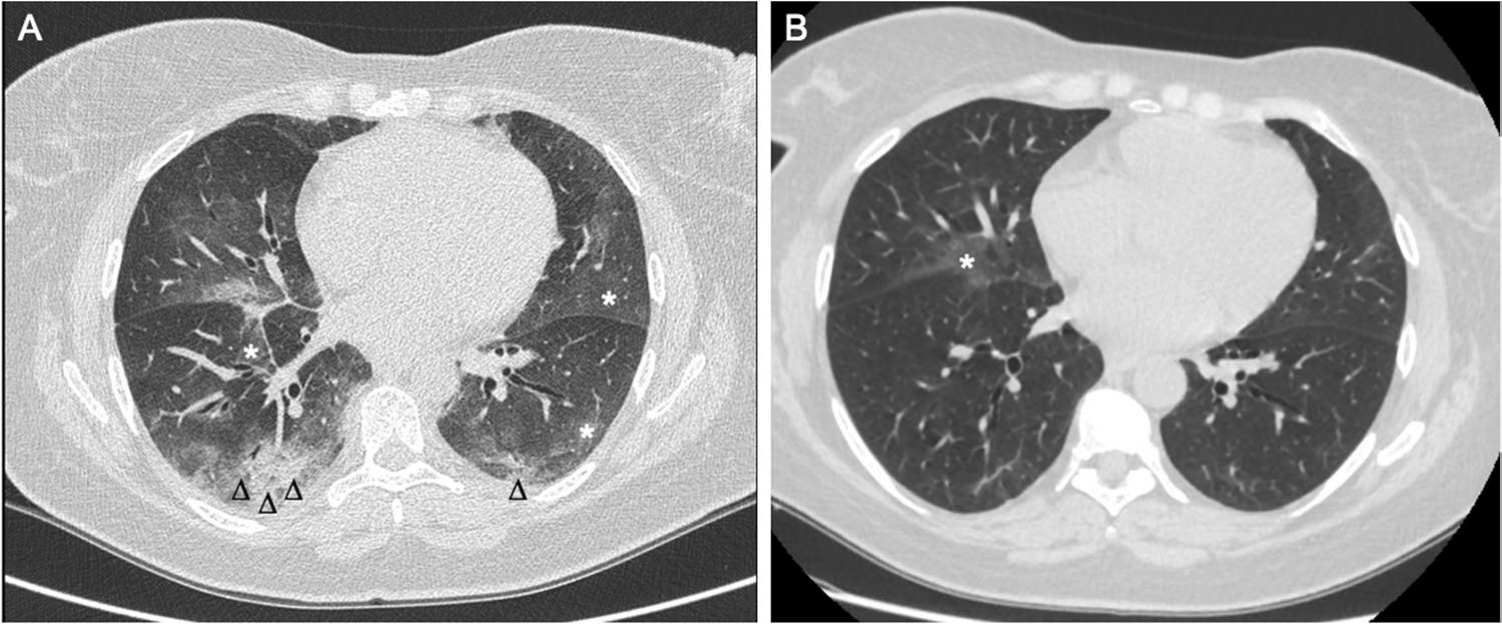
High-resolution computed tomography scans of a patient with rheumatoid arthritis-associated interstitial lung disease at baseline, starting abatacept treatment (**A**), and after 18 months of therapy (**B**). In **A** are detectable “ground-glass” opacities (asterisks) and pulmonary consolidations (arrowheads), while in **B** “ground-glass” opacities are significantly reduced and pulmonary consolidations disappeared

With regard to chest HRCT findings, significant changes in the CaM score were not detectable in the whole cohort (p = 0.71). At the end of the 18 months follow-up, 5 (11.4%) patients showed a HRCT deterioration of RA-ILD, 32 (72.6%) were considered stable, and 7 (16.0%) patients showed an HRCT improvement. Figure 1 shows an example of an improved patient. Analysing the differences between groups, the proportions of current smokers and of patients treated with MTX were significantly higher in the “worsened” compared to those defined as “improved/stable” (80% vs 33.35%, p = 0.01 and 60% vs 38.6%, p = 0.01, respectively).

At the multivariate regression analysis, the predictive variables related to RA-ILD progression during ABA therapy were concomitant treatment with MTX (p = 0.0078) and current smoking habit (p = 0.0054). Gender, disease duration, ACPA presence, RF presence, DLco, FVC, CDAI and HAQ-DI were not significantly associated with RA-ILD worsening (Table 2).

**Table 2:** Multivariate regression analysis of the variables predictive of pulmonary fibrosis evaluated at high-resolution computed tomography by the computer-aided method (dependent variable)

Independent variables	Coefficient	Standard error	t	p	r partial
(Constant)	1.6370				
Age (years)	-0.0012	0.0050	-0.238	0.8131	-0.0427
Gender	0.0254	0.1084	0.235	0.8161	0.0420
Disease duration (years)	0.0157	0.0176	0.891	0.3799	0.1580
ACPA positivity	-0.0129	0.0712	-0.182	0.8566	-0.0327
RF positivity	0.0030	0.0790	0.038	0.9694	0.0069
Current smokers	-0.3485	0.1165	-2.992	0.0054	-0.4733
Methotrexate use	-0.3956	0.1390	-2.847	0.0078	-0.4552
DLco (% predicted)	0.0052	0.0061	0.848	0.4030	0.1505
FVC (% predicted)	-0.0088	0.0088	-1.006	0.3224	-0.1777
CDAI	-0.0074	0.0038	-1.962	0.0588	-0.3324
HAQ-DI	-0.0173	0.1459	-0.119	0.9059	-0.0214

Abbreviations: ACPA = anti-cytrullinated protein antibodies; RF = rheumatoid factor; DLco = diffusion lung capacity of carbon monoxide; FVC = forced vital capacity; CDAI = Clinical Disease Activity Index; HAQ-DI = Health Assessment Questionnaire Disability Index

## Discussion

In this study it has been demonstrated that ABA plays a favourable role in the control of RA-ILD, with a significant worsening in only 11.4% of patients during the 18-month follow-up period.

This study consolidates some research already conducted regarding the value of ABA in RA-ILD. The first study on the safety of ABA in RA-ILD described four patients with RA-ILD exacerbated or manifested for the first time during treatment with csDMARDs. The patients were treated with ABA for a mean period of 35 months, without showing a worsening of lung function [20]. Nakashita and colleagues investigated the effect of ABA and other bDMARDs in two retrospective studies of RA-ILD patients [30, 31]. In the first study, they evaluated a group of patients starting therapy with bDMARDs and divided them into two groups according to the presence or absence of ILD at HRCT screening of the chest. After 12 months, patients who developed exacerbations or new ILD were all on TNFi therapy, whereas patients treated with ABA or tocilizumab had no exacerbations or onset of ILD in either group [30]. In the second study, patients with RA-ILD were evaluated, 16 of whom were starting ABA treatment and 46 of whom were starting TNFi treatment. After 12 months, ABA-treated patients showed no worsening of RA-ILD at chest HRCT, whereas 30% of TNFi-treated patients revealed a worsening of RA-ILD [31].

A Spanish multicentre retrospective study examined 63 RA-ILD patients, 15 of whom developed ILD immediately after the introduction of csDMARDs or bDMARDs. The authors investigated HRCT findings at baseline and after 12 months only in patients with persistent dyspnoea (22 patients, 34%). Of these, 50% showed stabilisation, 36.4% improvement and 13.6% worsening of ILD [19]. The same Spanish group recently expanded the multicentre study including 263 RA-ILD patients treated with ABA [21]. After 12 months of treatment, only 3 of 67 asymptomatic patients at baseline had mild dyspnoea, while 20% showed improvement in dyspnoea. FVC remained stable or improved in 87.7% of patients and DLCO in 90.6% of patients. HRCT improved in 24 cases (18.8%), while it worsened in 30 (23.4%) and the rest of patients remained stable. They also found a corticosteroid-sparing effect of ABA therapy.

This study is currently the one with the largest number of RA-ILD patients enrolled and treated with ABA. The obtained results show a clear majority of patients who “remain stable” or “improve” compared to those who “worsen”, thus affirming that ABA is a safe treatment in RA-ILD patients. Kurata and colleagues showed also that, in a RA population, after the initiation of bDMARD therapy, pre-existing airway disease is an independent risk factor for the onset or exacerbation of ILD, whereas ABA therapy is a protective factor [32]. ABA has a low-risk of worsening pre-existing ILD and could therefore play an important role in the clinical management of RA-ILD patients. We could speculate that ABA has a “protective effect” on the onset/exacerbation of ILD because, on one hand, the efficacy of blocking T-cell co-stimulation in the non-infectious lung inflammatory process has been demonstrated in the animal model of interstitial pneumonia; on the other hand, it is the drug with the lowest infectious risk compared to the other bDMARDs, thus causing a lower incidence of respiratory tract infections [18, 33, 34]. Regarding the latter, it is important to point out that other biotechnology drugs, recommended for RA patients with pre-existing ILD, have a high infectious risk and high rate of neutropenia or reduction of serum immunoglobulins [35, 36]. In fact, although there are no comparison studies, rituximab and tocilizumab treatments are associated with higher infectious risk than ABA in meta-analyses, and there are reports of exacerbations of persistent ILD or onset of ILD in RA patients on rituximab or tocilizumab therapy [37, 38]. In addition, further observations should be pointed out if we consider antifibrotic drugs in RA-ILD therapy. There are still some ongoing studies on this topic, but currently available data, extrapolated from some case reports, describe the successful use of nintedanib or pirfenidone in RA-ILD. Therefore, we believe that careful choice of therapy for RA-ILD patients is useful, particularly if a restrictive pulmonary syndrome is already present.

In the case of persistent active synovitis during ABA therapy, we suggest combining one or more DMARDs, preferably hydroxychloroquine or sulfasalazine for their low toxic effects on the lung if there are more than three inflamed joints, while it would be useful to undergo the patient to locoregional infiltrative therapy if there are two or fewer inflamed joints. In case of persistent active disease, it’s useful replacing the biotechnological drug as international guidelines suggest. For this reason, patients with high disease activity were excluded from our study.

In our cohort the percentage of “worsened” patients is higher than the evidence in the international literature, and this may be related to the different HRCT assessment method. To our knowledge, this is the first study using HRCT-CaM to estimate response to therapy in patients with RA-ILD. Recently we demonstrated that a quantitative analysis of ILD using a CaM is more responsive than applying a semi-quantitative visual method in assessing ILD progression in systemic sclerosis [27]. Therefore, the possibility of using standardised CaMs shared by the scientific community for HRCT quantification of ILD could be evaluated, both in research and clinical settings, due to the greater sensitivity in detecting differences [5] and more reliable results.

As a second goal, namely the identification of progression predictors for treatment response, current smokers and MTX-treated patients seemed to respond poorly to ABA. The relationship between cigarette smoking and respiratory disease is well documented. In addition, a strong causal relationship between cigarette smoking, the presence of ACPA and the development of RA-ILD has been widely demonstrated [39]. Therefore, all smoking patients should be helped to stop smoking because of its documented pulmonary toxicity.

In contrast, the relationship between MTX therapy and pulmonary response to ABA is challenging to explain [40]. MTX-induced pulmonary toxicity, presented as acute/subacute or rarely as chronic pneumonia, has been a subject of debate for many years [41, 42]. In recent years, however, this has been questioned with more emphasis on the increased risk of pulmonary infections in patients during MTX therapy rather than direct lung damage. In a meta-analysis of 21 studies from 1990 to 2011, including 8,276 RA patients, it was found that MTX was not associated with an increased risk of total adverse respiratory events and that there were no differences in the risk of pulmonary involvement between patients taking MTX and those not taking it. There was an increased risk of lower respiratory tract infection [43]. In clinical practice, distinguishing MTX-induced pulmonary toxicity from RA-ILD is a challenge and detecting the presence of an infection can be difficult. The worsening of ILD detected in the combined ABA and MTX patients in our study is therefore a fact of non-unique interpretation. Mochizuki and coworkers performed a retrospective study of 131 RA-patients treated with ABA and MTX, and they too found MTX to be a negative prognostic factor for lung response to ABA, as the RA-ILD patients who worsened were all on MTX-ABA combination therapy [44]. The most reasonable interpretation in light of recent data is that patients requiring ABA and MTX combination therapy have a more aggressive disease and may have more frequent extra-articular involvement. Although MTX is still the drug of choice for RA therapy, it may be advisable to reduce or discontinue it when clinical remission is achieved in RA-ILD patients, also in the light of international guidelines [45].

This scenario is also complicated by the presence of subclinical ILD in about 30% of RA patients, evaluated with chest HRCT [46, 47]. The lack of a valid screening tool to detect the presence of ILD in connective tissue diseases is another point of interest, due to the high exposure to ionising radiation of chest HRCT. A good correlation between HRCT and chest ultrasound in ILD detection has been demonstrated [48, 49], offering a valid and feasible tool for the detection of pulmonary involvement in RA patients [50], but not yet validated in international studies.

This study has some limitations: firstly, a low number of enrolled patients; secondly, a control group was not recruited; finally, we do not have data on the onset of ILD.

In conclusion, the use of ABA in the treatment of RA-ILD patients can be considered a first choice, especially for its proven safety and therefore for its likely efficacy on lung damage. It can also be stated that MTX therapy should be used with caution in patients with ILD due to the increased risk of infection.
